# Stress granules promote quiescence by enhancing p21 levels and reducing phospho-Rb

**DOI:** 10.1261/rna.080635.125

**Published:** 2025-10

**Authors:** Anthony Khong, Nina Ripin, Luisa Macedo de Vasconcelos, Victor Passanisi, Sabrina Spencer, Roy Parker

**Affiliations:** 1 Department of Biochemistry, BioFrontiers Institute, University of Colorado, Boulder, Colorado 80309, USA; 2 Howard Hughes Medical Institute, Chevy Chase, Maryland 20815, USA; 3 Cancer Science Institute of Singapore, National University of Singapore, Singapore 117599; 4 Department of Physiology, National University of Singapore, Singapore 117593

**Keywords:** stress granules, cell cycle, quiescence, G3BP, p21, vinorelbine

## Abstract

During the integrated stress response (ISR), most mRNAs exit translation and some condense into stress granules. Stress granules that form during chemotherapy can promote cancer cell survival and chemoresistance by an unknown mechanism. Cells can also spontaneously trigger the ISR at low levels, which promotes cellular quiescence where cells exit the cell cycle and are resistant to therapeutic agents. We hypothesized that the ability of cells to form stress granules might be a critical signal to drive cells into quiescence. Herein, we provide several observations that suggest stress granules enhance cell survival and chemoresistance by promoting cellular quiescence. The mechanism by which stress granules promote quiescence is by stimulating p21 expression, leading to inhibition of Rb phosphorylation. These results demonstrate that stress granule formation is sufficient to trigger cellular quiescence and argue that inhibitors of stress granules may be effective in combination with chemotherapy to limit the development of chemoresistance in treating human tumors.

## INTRODUCTION

Stress granules are cytosolic condensates that form under various cellular stress conditions, including oxidative stress, heat shock, osmotic stress, and chemotherapeutics (Anderson et al. 2015; Protter and Parker 2016). During cellular stress, the integrated stress response pathway is activated, which triggers eIF2α phosphorylation resulting in global translational initiation repression. Subsequently, most mRNAs are released from translating ribosomes resulting in the accumulation of nontranslating messenger ribonucleoproteins (mRNPs), which become the core assemblers and constituents of stress granules (Jain et al. 2016; Khong et al. 2017; Khong and Parker 2018; Markmiller et al. 2018; Namkoong et al. 2018; Youn et al. 2018).

Numerous studies indicate a close link between stress granules and cancer biology (Anderson et al. 2015; Song and Grabocka 2020; Lavalée et al. 2021). First, stress granules are found in various tumors (Somasekharan et al. 2015; Grabocka and Bar-Sagi 2016; Valentin-Vega et al. 2016; Attwood et al. 2020). Second, pro-tumorigenic signaling pathways promote stress granule assembly (Song and Grabocka 2020). Third, poor patient prognosis correlates with the high expression levels of many core stress granule assembly proteins (Somasekharan et al. 2015; Grabocka and Bar-Sagi 2016; Aucagne et al. 2017; Zhao et al. 2021). Fourth, many chemotherapeutic agents trigger stress granule formation (Fournier et al. 2010; Kaehler et al. 2014; Adjibade et al. 2015; Grabocka and Bar-Sagi 2016; Szaflarski et al. 2016; Bittencourt et al. 2019; Lin et al. 2019; Zhao et al. 2021). However, how stress granules contribute to cancer biology is not well understood. One of the prevailing models is that stress granules help cancer cells adapt to various cellular stress elicited by the tumor microenvironment and promote resistance to chemotherapeutic drugs (Anderson et al. 2015; Song and Grabocka 2020; Lavalée et al. 2021).

Several models have been proposed to explain how stress granules could contribute to cell survival during stress. One common theme in many of these models is the sequestration of proapoptotic proteins to stress granules leading to the dampening of apoptotic signaling pathways. This includes the proapoptotic proteins RACK1 (Arimoto et al. 2008), TRAF2 (Kim et al. 2005), RhoA–Rock1 complex (Tsai and Wei 2010), YWHAX (Zhao et al. 2021), BAX (Zhao et al. 2021), and mTORC1 constituents (Thedieck et al. 2013; Wippich et al. 2013). Another common theme is that stress granules may also promote cell survival by reducing reactive oxygen species through various mechanisms (Takahashi et al. 2013; Amen and Kaganovich 2021).

In this manuscript, we demonstrate that stress granules promote cell survival and chemoresistance by promoting cellular quiescence. Cellular quiescence is a state of reversible cell-cycle arrest with reduced transcription and translation from which cells can reenter the proliferative state. Cellular quiescence is thought to promote cell survival by reducing reactive oxygen species due to increased glycolysis over oxidative phosphorylation, upregulating antioxidant genes, increasing autophagic flux, and downregulating apoptosis genes (Coller et al. 2006; Marescal and Cheeseman 2020). Moreover, cellular quiescence also protects cells against chemotherapeutics, which generally target cycling cells.

In Min and Spencer (2019), the authors described a subpopulation of MCF10A cells that are slow-cycling. This subpopulation of cells frequently passes through a transient quiescent (G0) state in untreated conditions. This state is marked by low cyclin-dependent kinase 2 activity, high p21 levels, and hypophosphorylated Rb and is triggered by activation of stress-response pathways, including p53 activation, eIF2α phosphorylation, and the inhibition of translation. Since inhibition of translation should lead to the assembly of stress granules, we investigated if stress granules are involved in establishing or maintaining cellular quiescence.

## RESULTS

### Cells exhibiting spontaneous stress granules are often non-cycling

**FIGURE 1.**
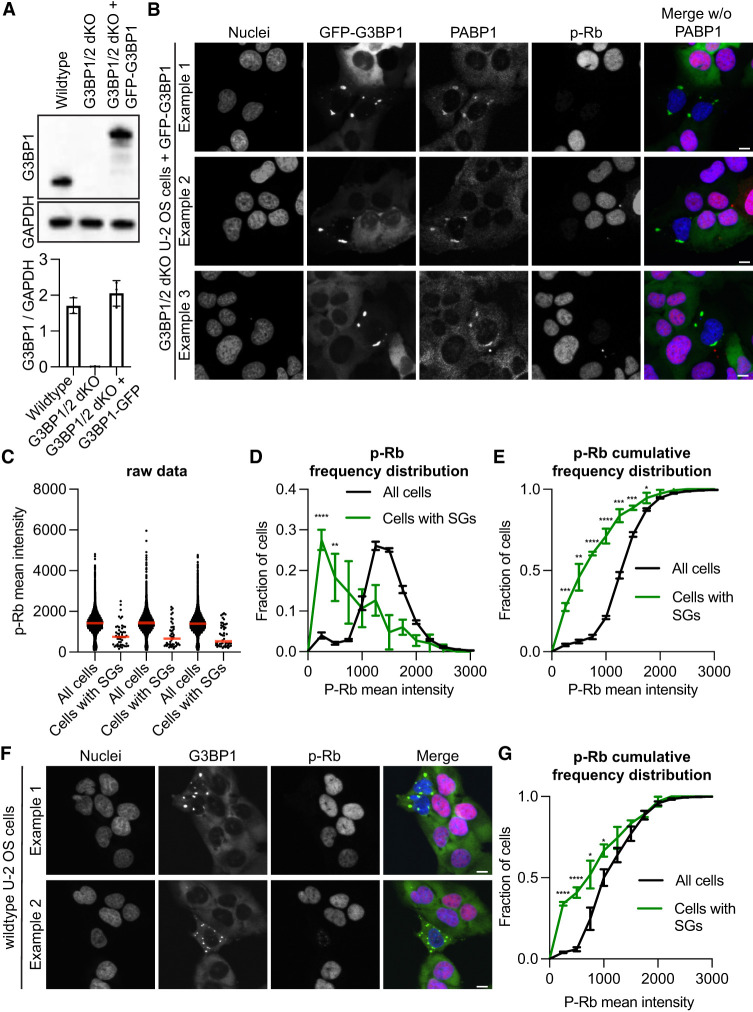
*G3BP1/2* genes promote a greater fraction of cells with low phospho-Rb (p-Rb) levels under non-stress conditions. (*A*, *top*) Representative immunoblot depicting the expression of G3BP1 and GAPDH in wild-type, *G3BP1/2* dKO U-2 OS and *GFP-G3BP1 + G3BP1/2* dKO U-2 OS cell lines. (*Below*) Box plot of G3BP1/GAPDH immunoblot intensities from three biological replicates. (*B*) Representative images illustrating *GFP-G3BP1 + G3BP1/2* dKO U-2 OS cells exhibiting spontaneous stress granules. 4',6-diamidino-2-phenylindole (DAPI) staining for nuclei, GFP fluorescence for GFP-G3BP1, PABP1 immunofluorescence, and p-Rb immunofluorescence are shown in grayscale individually. A merged image combines DAPI, GFP-G3BP1, and p-Rb channels in blue, green, and red, respectively, excluding PABP1. Scale bar, 20 µm. (*C*) Scatter plot illustrating the distribution of p-Rb levels in the nuclei of all cells and cells with observable stress granules for three independent biological replicates. Cells were considered to have stress granules when both GFP-G3BP1 and PABP1 were colocalized into puncta. The total number of cells and cells with stress granules for each replicate, respectively, are 10,552/54, 8237/48, and 9723/50. (*D*) Frequency distribution plot representing p-Rb intensity in the nuclei for all cells and cells with discernible stress granules in three biological replicates. (*E*) Cumulative frequency distribution plot illustrating p-Rb intensity in the nuclei for all cells and cells with visible stress granules in three biological replicates. (*F*) Representative images portraying wild-type U-2 OS cells with spontaneous stress granules. DAPI staining for nuclei, GFP fluorescence for GFP-G3BP1, and p-Rb immunofluorescence are shown in grayscale individually. Scale bar, 20 µm. (*G*) Cumulative frequency distribution plot illustrating p-Rb intensity in the nuclei for all cells and cells with evident stress granules for three biological replicates. Cells were considered to have stress granules if they contained G3BP1 puncta. The total number of cells and cells with stress granules for each replicate, respectively, are 4988/34, 4558/24, and 4667/27. (****) *P* < 0.0001, (***) *P* < 0.001, (**) *P* < 0.01, (*) *P* < 0.05.

If stress granules promote cells entering quiescence, stress granule formation should be observed in cells that spontaneously enter a quiescent state. To identify potentially quiescent cells, we used an antibody that recognizes Rb phosphorylation. Rb is hypophosphorylated in nonproliferating cells and becomes hyperphosphorylated upon cell-cycle commitment (Weinberg 1995). To visualize stress granules, we used a *G3BP1/2* double knockout (dKO) U-2 OS cell line, rescued with a *GFP-G3BP1* transgene via lentivirus transduction. This transgene is expressed at levels comparable to the endogenous G3BP1 proteins found in wild-type U-2 OS cells (Fig. 1A). Given the anticipated low frequency of spontaneous stress granules, we used a high-content screening microscope to rapidly image thousands of cells.

We observed that cells with spontaneous stress granules, identified by the colocalization of GFP-G3BP1 and PABP1 puncta, consistently exhibited reduced Rb phosphorylation (Fig. 1B–E). As anticipated, these spontaneous stress granules were observed in <1% of the population of *GFP-G3BP1 + G3BP1/2* dKO U-2 OS cells (Fig. 1B). To underscore the significance of the anticorrelation between p-Rb and stress granules in cells, we applied three distinct graphical analyses. Initially, raw p-Rb intensities were graphed for both all cells and cells with stress granules (Fig. 1C). Subsequently, we plotted a frequency distribution plot comparing p-Rb levels between all cells and the subset with stress granules (Fig. 1D). Finally, a cumulative frequency distribution plot of p-Rb was generated for all cells and those with stress granules (Fig. 1E). Collectively, these analyses consistently revealed a significant reduction in Rb phosphorylation in cells harboring stress granules. This finding indicates that cells with stress granules are frequently arrested or not actively engaged in the cell cycle in *the GFP-G3BP1 + G3BP1/2* dKO U-2 OS genetic background.

To expand the scope of our investigation beyond the ectopically expressed *GFP-G3BP1 + G3BP1/2* dKO U-2 OS cell, we examined whether wild-type U-2 OS cells with spontaneous stress granules were also withdrawn from the cell cycle. We discovered that wild-type U-2 OS cells do indeed develop spontaneous stress granules, evident through the presence of G3BP1 puncta by G3BP1 immunofluorescence, occurring in <1% of all cells (Fig. 1F). Our analysis revealed that these cells are frequently not engaged in the cell cycle, as indicated by our representative image and cumulative frequency plot (Fig. 1F,G). This finding provides further support that cells with spontaneous stress granules are often not actively participating in the cell cycle.

### G3BP1/2 deficiency reduces nonproliferating cells

In our previous analysis, we established a correlation between the presence of stress granules and cells that are not actively engaged in the cell cycle. To investigate the active role of stress granules in cell-cycle regulation, we postulated that cells lacking *G3BP1/2* genes should exhibit a reduced population of nonproliferating cells. To test this hypothesis, we conducted p-Rb staining experiments on wild-type U-2 OS cells, *G3BP1/2* dKO U-2 OS cells, and *GFP-G3BP1 + G3BP1/2* dKO U-2 OS cells to assess the differences in the proportion of cells not participating in the cell cycle.

**FIGURE 2.**
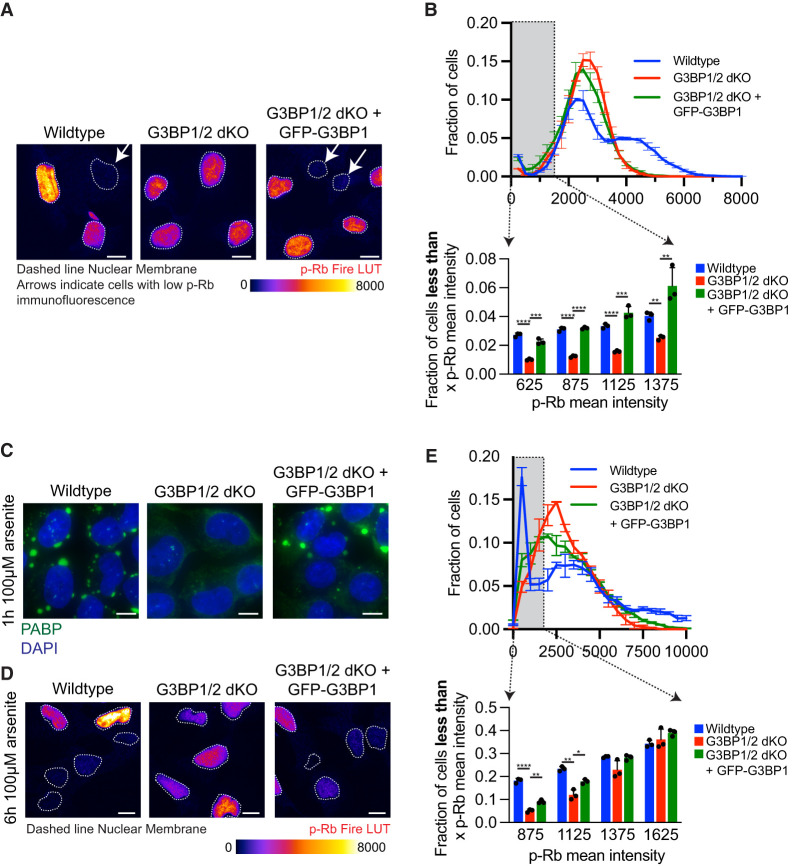
*G3BP1/2* genes promote cell-cycle exit. (*A*) Representative p-Rb immunofluorescence images of wild-type, *G3BP1/2* dKO, and *GFP-G3BP1 + G3BP1/2* dKO U-2 OS cells. Arrows indicate cells with low p-Rb immunofluorescence, while dashed line denotes the nuclear membrane. The immunofluorescence is presented in Fire look-up table (LUT) to enhance the visualization of differences in p-Rb levels. Scale bar, 10 µm. (*B*, *bottom*) Bar graph depicting the fraction of cells with mean p-Rb intensity in the nucleus below specified thresholds (625, 875, 1125, or 1375) for wild-type, *G3BP1/2* dKO, and *GFP-G3BP1 + G3BP1/2* dKO U-2 OS cells. Results are derived from the frequency distribution plot above where the fraction of cells is binned by p-Rb mean intensity levels in untreated wild-type, *G3BP1/2* dKO and *GFP-G3BP1 + G3BP1/2* dKO U-2 OS cells. Three independent biological replicates were conducted. (*C*) Representative PABP1 immunofluorescence (green channel) and DAPI stain (blue channel) images of wild-type, *G3BP1/2* dKO, and *GFP-G3BP1 + G3BP1/2* dKO U-2 OS cells treated with 100 µM arsenite for 1 h. Scale bar, 5 µm. (*D*) Representative p-Rb immunofluorescence images of wild-type, *G3BP1/2* dKO, and *GFP-G3BP1 + G3BP1/2* dKO U-2 OS cells in untreated conditions or treated with 100 µM arsenite for 6 h. The dashed line represents the nuclear membrane. The immunofluorescence is shown in Fire LUT to enhance the distinction in p-Rb levels between cells. Scale bar, 10 µm. (*E*, *bottom*) Bar graph illustrating the fraction of cells with mean p-Rb intensity in the nucleus below specified thresholds (625, 875, 1125, or 1375) for wild-type, *G3BP1/2* dKO, and *GFP-G3BP1 + G3BP1/2* dKO U-2 OS cells. Results are derived from the frequency distribution blot above where the fraction of cells are binned based on p-Rb mean intensity levels in 6 h 100 µM arsenite-treated, *G3BP1/2* dKO, and *GFP-G3BP1 + G3BP1/2* dKO U-2 OS cells. Three independent biological replicates were performed. (*) *P* < 0.05, (****) *P* < 0.0001, (***) *P* < 0.001, (**) *P* < 0.01.

Our investigation reveals cells deficient in *G3BP1/2* genes indeed exhibited fewer nonproliferating cells (Fig. 2A,B; Supplemental Fig. 1A,B). The representative images in Figure 2A visually demonstrate this decrease. To quantify our observations, we used three distinct metrics: raw data points of p-Rb intensities per nucleus (Supplemental Fig. 1A), frequency distribution of p-Rb levels per nucleus (Fig. 2B), and cumulative frequency of p-Rb values per nuclei (Fig. 2B; Supplemental Fig. 1B) across all three cell lines. The collective results from these analyses suggest that cells lacking *G3BP1/2* genes possess a diminished capacity to exit the cell cycle under non-stress conditions. This discovery indicates a role of *G3BP1/2* genes in regulating cellular quiescence and suggests a link between stress granules and cell-cycle control.

We extended our inquiry to stress conditions to determine if *G3BP1/2* genes are also involved in promoting and/or maintaining cell-cycle exit. Arsenite is known to robustly inhibit translation and induce stress granule formation (Kedersha et al. 1999). Treating cells with 100 µM arsenite for 60 min induces stress granules in wild-type and *GFP-G3BP1 + G3BP1/2* dKO U-2 OS cells, but not in *G3BP1/2* dKO U-2 OS cells (Fig. 2C; Supplemental Fig. 1C). Consistent with our previous findings regarding non-stress conditions, we observed a higher proportion of nonproliferating cells after 6 h of 100 µM arsenite treatment in wild-type and *GFP-G3BP1 + G3BP1/2* dKO U-2 OS cells compared to *G3BP1/2* dKO U-2 OS cells (Fig. 2D,E; Supplemental Fig. 1D,E). These results indicate that the G3BP1/2 proteins promote or maintain quiescence under stress conditions, further supporting a role of stress granules in cell-cycle regulation.

### *G3BP1/2* genes promote cell-cycle exit through stress granule formation

The role of *G3BP1/2* genes in facilitating cell-cycle exit gives rise to two not mutually exclusive possibilities: *G3BP1/2* foster cell-cycle exit through stress granules, and *G3BP1/2* promotes cell-cycle exit independent of stress of granules. To test the former hypothesis, we rescued stress granule assemblies in *G3BP1/2* dKO U-2 OS cells. If *G3BP1/2* genes indeed promote cell-cycle exit through stress granule formation, reintroducing stress granules in G3BP1/2 dKO U-2 OS cells should elevate the proportion of nonproliferating cells.

**FIGURE 3.**
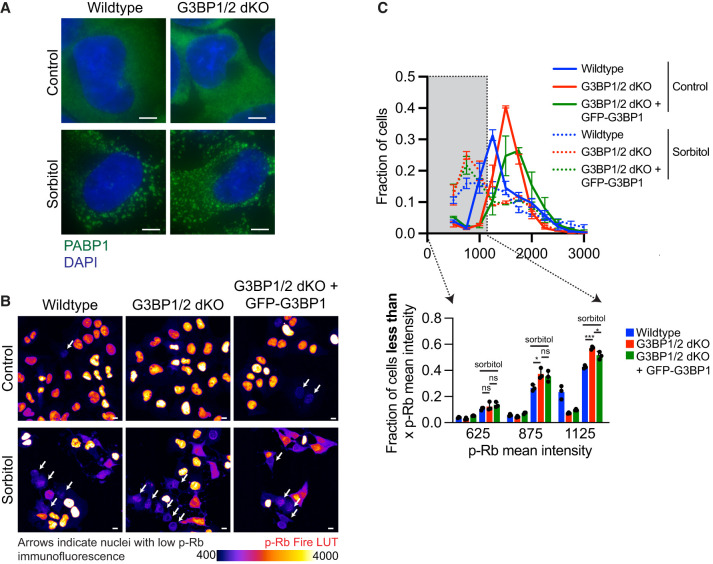
Sorbitol-induced stress granules promote cell-cycle exit in the absence of *G3BP1/2*. (*A*) Representative PABP1 immunofluorescence (green channel) and DAPI stain (blue channel) images of wild-type and *G3BP1/2* dKO under untreated conditions or treated with 0.5 M sorbitol for 1 h. Scale bar, 5 µm. (*B*) Representative p-Rb immunofluorescence images of wild-type, *G3BP1/2* dKO, and *GFP-G3BP1 + G3BP1/2* dKO U-2 OS cells under untreated conditions or treated with 0.5 M sorbitol for 6 h. The immunofluorescence is depicted using Fire LUT to accentuate differences in p-Rb levels between cells. Scale bar, 10 µm. (*C*, *bottom*) Bar graph illustrating the fraction of cells with mean p-Rb intensity in the nucleus below specified thresholds (625, 875, and 1125) for wild-type, *G3BP1/2* dKO, and *GFP-G3BP1 + G3BP1/2* dKO U-2 OS cells from the experiment shown in *B*. The bar graph is derived from the frequency distribution blot above which represents the fraction of cells categorized based on binned p-Rb mean intensity levels in untreated and 0.5 M 6 h sorbitol-treated wild-type, *G3BP1/2* dKO and *GFP-G3BP1 + G3BP1/2* dKO U-2 OS cells. Three independent biological replicates were conducted.

Initially, we induced stress granule assembly in wild-type U-2 OS, *G3BP1/2* dKO U-2 OS, and *GFP-G3BP1 + G3BP1/2* dKO cells using 0.5 M sorbitol, a stressor known to induce stress granules independently of *G3BP1/2* genes (Fig. 3A; Supplemental Fig. 2A). After exposing these cell lines to 0.5 M sorbitol stress for 6 h, we stained the cells for p-Rb and quantified the level of cells that are nonproliferating (Fig. 3B,C; Supplemental Fig. 2B,C). We observed a comparable percentage of hypophosphorylated Rb cells between wild-type, *G3BP1/2* dKO, and *GFP-G3BP1 + G3BP1/2* dKO U-2 OS cells under sorbitol stress (Fig. 3B,C; Supplemental Fig. 2B,C). These findings support the idea that stress granule formation itself, independent of *G3BP1/2* genes, drives cell-cycle exit.

**FIGURE 4.**
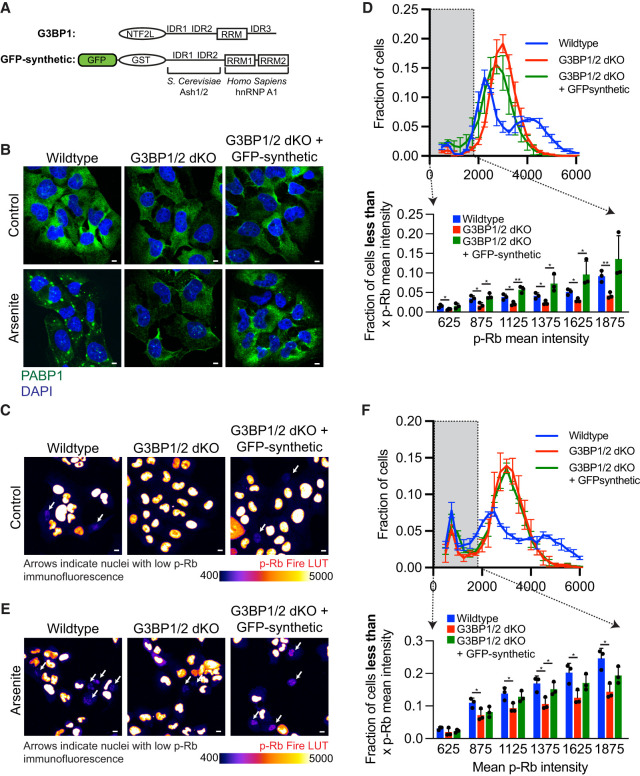
*GFP-synthetic* gene promotes cell-cycle exit in the absence of *G3BP1/2*. (*A*) Cartoon schematic depicting the protein domains of G3BP1 and GFP-synthetic. (*B*) Representative PABP1 immunofluorescence (green channel) and DAPI stain (blue channel) images of wild-type, *G3BP1/2* dKO, and *GFP-synthetic + G3BP1/2* dKO U-2 OS cells under untreated conditions or treated with 100 µM arsenite for 1 h. Scale bar, 5 µm. (*C*) Representative p-Rb immunofluorescence images of wild-type, *G3BP1/2* dKO, and *GFP-synthetic + G3BP1/2* dKO U-2 OS cells in untreated conditions. The immunofluorescence is presented in Fire LUT to emphasize differences in p-Rb levels between cells. Scale bar, 10 µm. (*D*, *bottom*) Bar graph illustrating the fraction of cells with mean p-Rb intensity in the nucleus below specified thresholds (625, 875, 1125, 1375, 1625, and 1875) for wild-type, *G3BP1/2* dKO, and *GFP-synthetic + G3BP1/2* dKO U-2 OS cells from the experiment shown in *C*. The bar graph was derived from a frequency distribution blot displayed above based on the fraction of cells binned by p-Rb mean intensity levels in untreated wild-type, *G3BP1/2* dKO, and *GFP-synthetic + G3BP1/2* dKO U-2 OS cells. Three independent biological replicates were performed. (*E*) Representative p-Rb immunofluorescence images of wild-type, *G3BP1/2* dKO, and *GFP-synthetic + G3BP1/2* dKO U-2 OS cells under 6 h 100 µM arsenite-treated conditions. The immunofluorescence is depicted using the Fire LUT to enhance the distinction in in p-Rb levels between cells. Scale bar, 10 µm. (*F*, *bottom*) Bar graph illustrating the fraction of cells with mean p-Rb intensity in the nucleus below specified thresholds (625, 875, 1125, 1375, 1625, and 1875) for wild-type, *G3BP1/2* dKO, and *GFP-synthetic + G3BP1/2* dKO U-2 OS cells from the experiment shown in *E*. The bar graph was derived from a frequency distribution blot displayed above based on fraction of cells binned by p-Rb mean intensity levels in 6 h 100 µM arsenite-treated wild-type, *G3BP1/2* dKO and *GFP-synthetic + G3BP1/2* dKO U-2 OS cells. Three independent biological replicates were performed. (ns) *P* > 0.05, (*) *P* < 0.05, (****) *P* < 0.0001, (***) *P* < 0.001, (**) *P* < 0.01.

In a separate stress granule rescue experiment, we reintroduced stress granule formation in *G3BP1/2* dKO U-2 OS cells using an engineered gene termed *GFP-synthetic*, designed to mimic G3BP1 protein multivalency and architecture but with all protein domains replaced (Fig. 4A). This artificial gene was effective in restoring stress granules in *G3BP1/2* dKO U-2 OS cells under arsenite stress, albeit a reduced rate, validating previous findings (Fig. 4B; Supplemental Fig. 3A; Yang et al. 2020).

Using this system, we examined whether *GFP-synthetic* could mimic the proportion of nonproliferating cells in *G3BP1/2* dKO U-2 OS cells. Strikingly, under both non-stress conditions and arsenite-induced stress, the introduction of *GFP-synthetic* transgene successfully increased the levels of nonproliferating cells in *G3BP1/2* dKO U-2 OS cells (Fig. 4C–F; Supplemental Fig. 3B–E). These results provide further evidence that stress granule formation itself, independent of *G3BP1/2* genes, is a driver of cell-cycle exit.

### Stress granules induce cellular quiescence by promoting p21 mRNA expression

**FIGURE 5.**
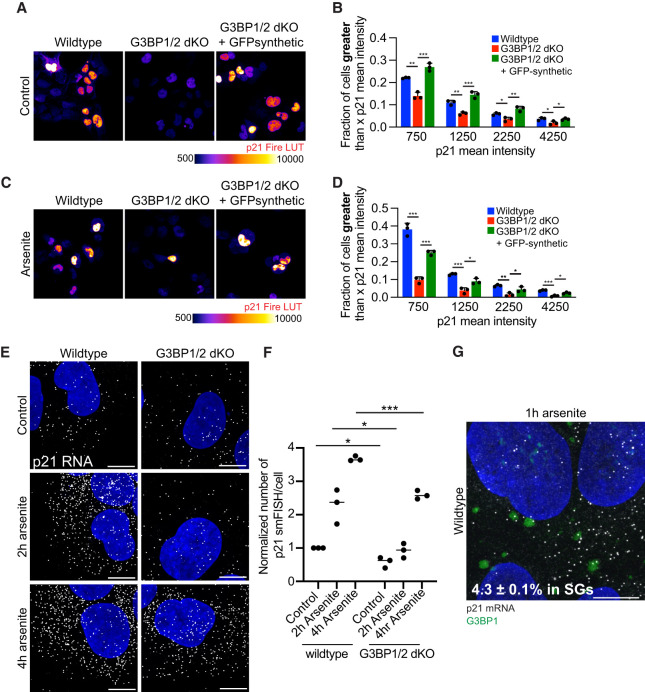
Stress granules promote p21 expression under non-stress and stress conditions. (*A*) Representative p21 immunofluorescence images of untreated wild-type, *G3BP1/2* dKO, and *GFP-G3BP1 + G3BP1/2* dKO U-2 OS cells. The p21 immunofluorescence is color-coded using the Fire LUT for enhanced visualization of variations in p21 levels among cells. Scale bar, 10 µm. (*B*) Bar graph illustrating the fraction of cells with mean p21 intensity in the nucleus above specified thresholds (750, 1250, 2250, and 4250) for wild-type, *G3BP1/2* dKO, and *GFP-synthetic + G3BP1/2* dKO U-2 OS cells from the experiment shown in *A*. Three independent biological replicates were conducted. (*C*) Representative p21 immunofluorescence images of wild-type, *G3BP1/2* dKO, and *GFP-G3BP1 + G3BP1/2* dKO U-2 OS cells treated with 100 µM arsenite for 6 h. The p21 immunofluorescence is color-coded using the Fire LUT for visualization of variations in p21 levels between cells. Scale bar, 10 µm. (*D*) Bar graph illustrating the fraction of cells with mean p21 intensity in the nucleus above specified thresholds (750, 1250, 2250, and 4250) for arsenite-treated wild-type, *G3BP1/2* dKO, and *GFP-synthetic + G3BP1/2* dKO U-2 OS cells from the experiment shown in *C*. Three independent biological replicates were performed. (*E*) Representative *p21* mRNA single-molecule fluorescence in situ hybridization (FISH) (white) and DAPI (blue) images of wild-type and *G3BP1/2* dKO U-2 OS cells that were either untreated (control) or treated for 2 or 4 h with 200 µM arsenite. (*F*) Scatterplot of the average number of *p21* mRNA transcripts per cell from *E*. Three independent biological replicates were performed. (*G*) Representative *p21* single-molecule FISH (white), G3BP1 immunofluorescence images (green), and DAPI (blue) of wild-type U-2 OS cells that were treated for 1 h with 200 µM arsenite. The percentage indicates the average number of *p21* mRNAs in stress granules across three biological replicates. Scale bar, 10 µm. (ns) *P* > 0.05, (*) *P* < 0.05, (**) *P* < 0.01.

Rb phosphorylation, cell-cycle exit, and quiescence are regulated by many cyclin-dependent kinase inhibitors, with a critical regulator being p21, an inhibitor of CDK2–Cyclin A/E complexes. Therefore, we investigated if stress granules increased p21 levels by examining the levels of p21 proteins in wild-type, *G3BP1/2* dKO, and *GFP-synthetic + G3BP1/2* dKO U-2 OS in individual cells by immunofluorescence under non-stress (Fig. 5A,B) and arsenite-stress conditions (Fig. 5C,D).

We observed that *G3BP1/2* dKO cells contain fewer cells with high p21 protein levels under non-stress and arsenite conditions (Fig. 5A–D; Supplemental Fig. 4A–F), indicating that stress granules promote cellular quiescence by increasing p21 expression.

To dissect the underlying mechanism, we further examined *p21* mRNA levels. Using single-molecule FISH and RNA sequencing, we compared *p21* mRNA levels between wild-type and *G3BP1/2* dKO U-2 OS cells. Significantly lower *p21* mRNA counts per cell were consistently observed in *G3BP1/2* dKO U-2 OS cells under non-stress conditions and during 2 and 4 h arsenite stress (Fig. 5E,F).

One possibility is that the *p21* mRNA is sequestered in stress granules, where it could be stabilized by RNA binding proteins (Gareau et al. 2011). However, by both single-molecule FISH (Fig. 5G) and RNA-seq of purified stress granules (Khong et al. 2017), we observed that only a small fraction (4.3% by single-molecule FISH and 4% by RNA-seq) of the *p21* mRNAs are recruited to stress granules during arsenite stress (Fig. 5G; Khong et al. 2017). This implies that *p21* mRNA is not regulated by sequestering to stress granules and suggests stress granule formation might regulate *p21* mRNA levels indirectly.

### G3BP1/2 promote cellular quiescence in response to chemotherapeutics

**FIGURE 6.**
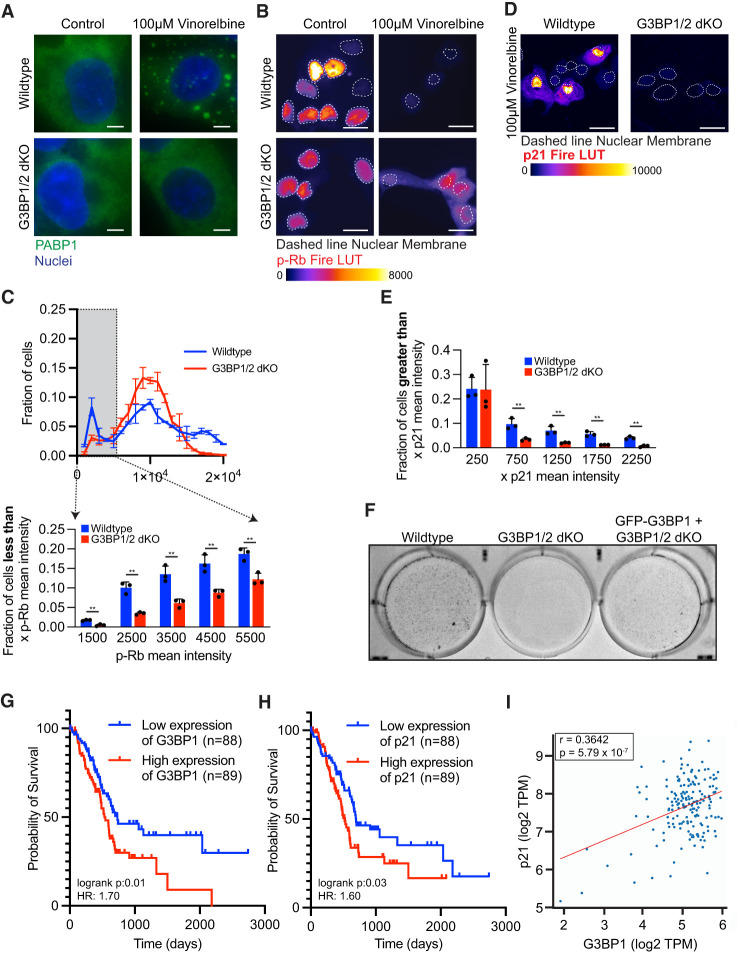
*G3BP1/2* genes promote a greater fraction of cells with low p-Rb levels and greater levels of chemoresistance when treated with vinorelbine. (*A*) Representative PABP1 immunofluorescence images of wild-type and *G3BP1/2* dKO U-2 OS cells treated with 100 µM vinorelbine or water (control) for 1 h. Scale bar, 5 µm. (*B*) Representative p-Rb immunofluorescence images of wild-type and *G3BP1/2* dKO U-2 OS cells when treated with 100 µM vinorelbine or water (control) for 6 h. p-Rb immunofluorescence is color-coded using the Fire LUT, and the dashed lines indicate the nuclear membrane. Scale bar, 10 µm. (*C*, *bottom*) Bar graph illustrating the fraction of cells with mean p-Rb intensity in the nucleus below specified thresholds (1500, 2500, 3500, 4500, and 5500) for vinorelbine-treated wild-type and G3BP1/2 dKO from the experiment shown in *B*. The bar graph is derived from the frequency distribution blot above where the fraction of cells is binned by p-Rb mean intensity levels in vinorelbine-treated wild-type and *G3BP1/2* dKO U-2 OS cells. Three independent biological replicates were performed. (*D*) Representative p21 immunofluorescence images of wild-type and *G3BP1/2* dKO U-2 OS cells treated with 100 µM vinorelbine or water (control) for 6 h. The p21 immunofluorescence is color-coded using the Fire LUT, and dashed lines indicate the nuclear membrane. Scale bar, 25 µm. (*E*) Bar graph illustrating the fraction of cells with mean p21 intensity in the nucleus above specified thresholds (250, 750, 1250, and 2250) for vinorelbine-treated wild-type and *G3BP1/2* dKO U-2 OS cells from the experiment shown in *D*. Three independent biological replicates were performed. (*F*) Representative image of the colony formation of wild-type, *G3BP1/2* dKO, and *GFP-G3BP1 + G3BP1/2* dKO U-2 OS cells stained with 0.5% crystal violet. Cells treated with 70 µM vinorelbine for 3 h, washed, and trypsinized, and 100,000 cells were seeded in a new plate. After 12 days of recovery, colony formation was assessed. The image is one representative biological replicate out of three. (*G*) Progression-free survival curves comparing high and low expression of *G3BP1* in pancreatic adenocarcinoma patients sourced from OncoDB using The Cancer Genome Atlas (TCGA) data sets. (*H*) Progression-free survival curves comparing high and low expression of *p21* in pancreatic adenocarcinoma patients from OncoDB using TCGA data sets. (*I*) Pair-wise gene expression correlation between *G3BP1* and *p21* genes in pancreatic adenocarcinoma tumors sourced from OncoDB using TCGA data sets. Graphs were retrieved from http://OncoDB.org.

Since cellular quiescence can facilitate chemoresistance (Min and Spencer 2019; Recasens and Munoz 2019), we examined if *G3BP1/2* affected chemoresistance to 100 µM vinorelbine for 6 h, which induces stress granules robustly in wild-type but not in *G3BP1/2* dKO U-2 OS cells after 1 h (Fig. 6A; Szaflarski et al. 2016). Importantly, we observed fewer quiescent cells in *G3BP1/2* dKO U-2 OS cells compared to wild-type cells (Fig. 6B,C; Supplemental Fig. 5A,B). Concurrently, we see more cells with high p21 expression in wild-type cells compared to *G3BP1/2* dKO U-2 OS cells (Fig. 6D,E; Supplemental Fig. 5C–E). These results suggest stress granule–induced quiescence in response to vinorelbine requires stress granule formation.

We hypothesized that stress granule formation, and the formation of quiescent cells, would increase the survival of a small fraction of cells when treated with chemotherapeutics, and those cells would regrow after the cessation of chemotherapy, which would be analogous to the regrowth of a pool of quiescent tumor cells after ceasing a round of chemotherapy. To test this prediction, we subjected wild-type, *G3BP1/2* dKO, and *GFP-G3BP1 + G3BP1/2* dKO U-2 OS cells to 3 h of 70 µM vinorelbine, washed out the chemotherapeutic, seeded the same number of cells in a new plate, and examined if cells could recover and grow into colonies after 10 days.

A striking result was that wild-type U-2 OS cells and *GFP-G3BP1 + G3BP1/2* dKO U-2 OS cells formed many more colonies than *G3BP1/2* dKO cells (Fig. 6F; Supplemental Fig. 5F). These findings indicate vinorelbine can trigger stress granules, which may promote cellular quiescence and chemoresistance.

### Expression of stress granule proteins is correlated with higher p21 expression in pancreatic ductal adenocarcinoma

To determine if the role of stress granules in promoting quiescence was relevant to tumor progression, we examined how the expression of key stress granule components correlated with *p21* expression, and prognosis, in patients using data sets from OncoDB, an online database containing clinical data of gene expression in cancer from TCGA (Tang et al. 2022). We focused on pancreatic adenocarcinoma, since this cancer is known to have stress granules in vivo (Grabocka and Bar-Sagi 2016; Fonteneau et al. 2022; Redding et al. 2025). We observed that patients have a poorer clinical outcome when tumors are expressing high levels of positive regulators of stress granules *G3BP1* (Fig. 6G), *G3BP2* (Supplemental Fig. 6A), *CAPRIN1* (Supplemental Fig. 6B), and *p21* (Fig. 6H). Moreover, we observed a correlation between the expression of *G3BP1* and *p21* (Fig. 6I), *G3BP2* and *p21* (Supplemental Fig. 6C), and *CAPRIN1* and *p21* (Supplemental Fig. 6D). These findings suggest that stress granules may promote *p21* expression in pancreatic adenocarcinoma tumors, contributing to the highly refractory chemoresistance of pancreatic adenocarcinoma.

## DISCUSSION

We provide several observations demonstrating stress granule formation promotes entry into quiescence, which may contribute to chemoresistance. First, cells with spontaneous stress granules are often quiescent (Fig. 1B–G). Second, cells deficient in stress granule assembly (*G3BP1/2* dKO) contain fewer quiescent cells under both non-stress, arsenite, and vinorelbine conditions (Figs. 2A–E, 6A–C; Supplemental Figs. 1A–E, 5A,B). Third, rescuing stress granule formation with 0.5 M sorbitol or *GFP-synthetic* in the *G3BP1/2* dKO U-2 OS restores the levels of cellular quiescence similar to wild-type U-2 OS cells (Figs. 3A–C, 4A–F; Supplemental Figs. 2A–C, 3A–E). Fourth, cells deficient in G3BP1/2 proteins, which contain fewer quiescent cells, are more sensitive to vinorelbine toxicity and recover less efficiently by forming fewer colonies (Fig. 6F; Supplemental Fig. 5F). Sixth, patients with pancreatic adenocarcinoma tumors show a correlation between high levels of stress granule proteins, poor prognosis, and increased p21 levels (Fig. 6G–I; Supplemental Fig. 6A–D). We interpret these results to demonstrate that stress granule formation promotes entry into a quiescent state, which may drive chemoresistance.

Three types of observations suggest that the formation of cellular quiescence by stress granules may have broad significance. First, cellular quiescence promotes resistance to many chemotherapeutic agents (Naumov et al. 2003; Dembinski and Krauss 2009; Chen et al. 2012; Brown et al. 2017; Min and Spencer 2019). Second, stress granule formation is triggered by many different types of chemotherapeutics, including alkylating agents, antimetabolites, mitotic inhibitors, kinase inhibitors, and proteasome inhibitors (Fournier et al. 2010; Kaehler et al. 2014; Adjibade et al. 2015; Grabocka and Bar-Sagi 2016; Szaflarski et al. 2016; Bittencourt et al. 2019; Lin et al. 2019; Zhao et al. 2021). Third, many of the other mechanisms by which stress granules have been proposed to enhance chemoresistance, including inhibiting apoptosis (Kim et al. 2005; Arimoto et al. 2008; Thedieck et al. 2013), reducing reactive oxygen species (Takahashi et al. 2013; Amen and Kaganovich 2021), and decreasing metastasis (Somasekharan et al. 2015), are also properties of quiescent cells (Coller et al. 2006; Goddard et al. 2018; Marescal and Cheeseman 2020).

Besides promoting chemoresistance, the ability of stress granules to induce cellular quiescence may enhance metastasis. Dormant cancer cells can metastasize to distant areas, remain quiescent for up to 20 years, and reenter the cell cycle (Goddard et al. 2018). Quiescence is thought to facilitate metastasis by aiding cancer cells to survive in new local environments. Strikingly, *G3BP1/2* genes are required for efficient metastasis in some xenograft models (Somasekharan et al. 2015). Moreover, recent studies suggest treating tumors with chemotherapeutics, which should promote stress granule formation, also promotes metastasis (D'Alterio et al. 2020). These results suggest the importance of studying stress granules in metastasis, and chemotherapeutic treatments should avoid triggering the assembly of stress granules.

One major unresolved question is how p21 expression is regulated by stress granules. Gareau et al. (2011) indicate stress granules can regulate *p21* stability under bortezomib stress in HeLa cells by stabilizing the normally unstable *p21* mRNAs through sequestration to stress granules (Gareau et al. 2011). We only saw a minor fraction of p21 mRNAs recruited to stress granules under arsenite stress in U-2 OS cells (Fig. 5G). This argues that stress granules regulate *p21* mRNA expression either by promoting *p21* transcription, and/or indirectly promoting *p21* mRNA stability by the activation of cellular signaling pathways, several of which are proposed to be regulated by stress granules in this manner (Arimoto et al. 2008; Tsai and Wei 2010; Thedieck et al. 2013; Wippich et al. 2013).

Recognizing the importance of cellular quiescence in tumors, new therapies are being developed to “lock out” cancer cells from cellular quiescence, which renders the tumors susceptible to chemotherapeutics and also limits metastasis (Recasens and Munoz 2019). Therefore, inhibiting stress granules may provide another therapeutic strategy to “lock out” cancer cells from cellular quiescence. Moreover, elucidating the mechanisms of how stress granules promote quiescence may also reveal new molecular targets for limiting cellular quiescence and treating tumors.

### Limitations of the study

We acknowledge several limitations in this study. First, our analysis was restricted to a single cancer cell line, U-2 OS, making it uncertain whether the findings can be generalized across other cancer models. Second, we assessed cell-cycle exit using only two markers, p-Rb and p21, which may not fully represent the complexity of this process. Additional cell-cycle markers such as Ki67 and EdU would provide further support of the major findings in this manuscript. Third, it is possible that stress granules may extend the duration spent in quiescence, which we did not examine (rather than promoting exit from cell cycle); while our p21 data support a role in quiescence entry, we cannot exclude the possibility that stress granules contribute to both initiation and maintenance of the quiescent state. Fourth, this study only rescues *G3BP1* in *G3BP1/2* dKO cells. It is unclear if *G3BP2* can phenocopy *G3BP1* in terms of rescuing p-Rb and should be evaluated in the future. Fifth, the distribution of p-Rb levels can vary a lot across cell lines and cannot be fully rescued in *G3BP1/2* dKO cells with *G3BP1* transgene. This could be due to a variety of factors including CRISPR-generated clone artifacts. *G3BP2* may also be required to restore full p-Rb distribution profile, and other factors such as exogenously introduced *G3BP1* transgene are likely not epigenetically regulated like endogenous *G3BP1* gene. Sixth, the concentration of vinorelbine used in our experiments exceeded *C*_max_ values of 130 ng/mL (or 120 nM) (AusPAR Navelbine Pierre Fabre Medicament, Australia 2005), potentially impacting the translatability of our results. Seventh, our correlation analysis between *G3BP1/2* expression and patient survival in pancreatic ductal adenocarcinoma (PDAC) tumors is inherently limited by its observational nature; it establishes correlation, not causation, and does not exclude the possibility that non-stress granule functions of *G3BP1/2* could influence patient survival. Lastly, and most importantly, our study does not elucidate the molecular mechanism by which stress granules promote cell-cycle exit. Identifying this mechanism would significantly strengthen our findings, and we plan to address these limitations in future studies.

## MATERIALS AND METHODS

### Cell lines

Wild-type and *G3BP1/2* dKO U-2 OS were kindly provided to us by Paul Anderson's lab at Brigham Children's Hospital (Kedersha et al. 2016). The *GFP-G3BP1 + G3BP1/2* dKO U-2 OS cell line was generated in the Parker lab using a lentivirus containing GFP-G3BP1 gene that was established by James Burke (Burke et al. 2020). The *GFP-synthetic + G3BP1/2* dKO U-2 OS cell line was generated in the Parker lab using a lentivirus containing *GFP-synthetic gene*. This construct was established by subcloning “GFP-synthetic” from pEGFP-C3 (Yang et al. 2020) to pRP2988 lentiviral transfer plasmid (Burke et al. 2020).

### Cell's growth conditions

U-2 OS cells were maintained in Dulbecco's modified Eagle medium (DMEM) with 10% fetal bovine serum (FBS) and 1% penicillin/streptomycin at 37°C/5% CO_2_.

### Chemicals and small molecules

Wild-type, *G3BP1/2* dKO U-2 OS, and *GFP-G3BP1 + G3BP1/2* dKO U-2 were treated with various compounds at various concentrations and time, as noted in each figure. The following compounds were used: sodium (meta) arsenite (Millipore Sigma; S7400-100G), vinorelbine ditartrate (Tocris; 3401), and D-sorbitol (Millipore Sigma; S1876).

### Colony forming assay

A total of 200,000 wild-type or *G3BP1/2* dKO U-2 OS cells were seeded in 6-well plates. Cells were then treated with 70 µM vinorelbine for 3 h, washed twice with phosphate buffered saline (PBS), and incubated with 0.05% trypsin for 5 min. Cells were then spun down into a pellet at 1000*g* and resuspended in 1 mL of media. Cells were then counted, and 100,000 cells were added to a new well in a 6-well plate in 2 mL of media and incubated for 10 days at 37°C/5% CO_2_ to allow cells to grow into large colonies. Colonies were then washed twice with cold PBS and fixed with cold methanol for 10 min. Colonies were then stained with 0.5% crystal violet solution (Thermo Fisher Scientific; S25275B) for 10 min and washed with water until the residue dye came off.

### Immunoblotting

Cells were washed with ice-cold PBS and lysed in Pierce RIPA Buffer (Thermo Fisher Scientific; 89900), 1 mM dithiothreitol (DTT), and cOmplete, Mini, EDTA-free Protease Inhibitor Cocktail (Sigma-Aldrich; 11836170001) on ice for 10 min and then clarified by centrifugation (13K RPM for 10 min). The 4×SDS sample loading buffer (Thermo Fisher Scientific; NP0007) was added to lysates to a final concentration of 1×; samples were boiled for 5 min at 95°C, then loaded into 4%–12% Bis-Tris NuPAGE gel (Thermo Fisher Scientific; NP0322BOX), and transferred to a nitrocellulose membrane. Membranes were blocked with 5% nonfat-dried milk in Tris-buffered saline with 0.1% Tween-20 (TBST) and then incubated with 1:1000 primary mouse anti-G3BP1 antibody in 5% nonfat-dried milk TBST (Abcam; ab56574) for 1 h followed by 3× TBST washes and incubation with 1:1000 in 5% nonfat-dried milk TBST peroxidase (HRP) anti-mouse IgG horse secondary antibody (Cell Signaling Technology; 7076S). Blots were stripped using Thermo Scientific Restore PLUS Western Blot Stripping Buffer (Thermo Fisher Scientific; 46430) by incubating for 15 min at room temperature. For loading control, membranes were incubated at room temperature for 30 or 60 min with the 1:1000 HRP conjugated anti-GAPDH in 5% nonfat-dried milk TBST (Santa Cruz Biotechnology; sc-47724). After 3× final TBST washes, antibody detection was achieved by applying Pierce ECL western blotting substrate for 5 min on the membranes and imaged on iBright 1500 (Invitrogen; A44114).

### Immunofluorescence

#### Analysis of individual phospho-Rb and p21 nuclear intensities

A total of 10,000 U-2 OS cells were seeded in CellCarrier-96 Ultra Microplates (PerkinElmer; 6055300), or 50,000 U-2 OS cells were seeded in 24-well glass bottom plates (Cellvis; P24-1.5H-N) and incubated overnight at 37°C/5% CO_2_. Cells were either untreated or treated with various compounds for 6 h as noted. Cells were then washed once with PBS, fixed with 4% paraformaldehyde (Santa Cruz Biotechnology; sc-281692) for 5 min, and permeabilized with 0.1% Triton X-100 in PBS for 5 min. Cells were stained and incubated with 1:250 primary rabbit antibody anti-phospho-Rb (Cell Signaling Technology; 8516) or 1:250 primary rabbit antibody anti-p21 Waf1/Cip1 (12D1) (Cell Signaling Technology; 2947S) for 1 h. Cells were then washed 3× with PBS. Subsequently, cells were stained with goat anti-rabbit Alexa Fluor 657 (Abcam; ab150079). Cells were washed 3× with PBS, stained for 5 min with 1:2000 DAPI (Thermo Fisher Scientific; 62248) in PBS, washed 2× with PBS, and stored in PBS at 4°C.

#### Analysis of stress granules

A total of 200,000 U-2 OS cells were seeded in 6-well plates containing EtOH-treated coverslips and incubated overnight at 37°C/5% CO_2_. Cells were then either untreated or treated with various compounds for 1 h, as noted. Cells were then washed once with PBS, washed three times with PBS, fixed with 4% paraformaldehyde (Santa Cruz Biotechnology; sc-281692) for 5 min, and permeabilized with 0.1% Triton X-100 in PBS for 5 min. Cells were stained and incubated with primary antibodies (1:1000 rabbit anti-PABP [Abcam; ab21060] and/or 1:1000 mouse anti-G3BP1 antibody [Abcam; ab56574]) for 1 h. Cells were then washed 3× with PBS. Subsequently, cells were stained with secondaries (goat anti-rabbit FITC [Abcam; ab6717] or goat anti-rabbit Alexa Fluor 647 [Abcam; ab150079]). Cells were washed 3× with PBS and stained for 5 min with 1:2000 DAPI (Thermo Fisher Scientific; 62248) in PBS. Cells were then washed 2× with PBS, and the coverslips were mounted on slides with VECTASHIELD Antifade Mounting Medium (Vector Laboratories; H-1000).

#### Analysis of *p21* mRNAs by single-molecule FISH

The single-molecule protocol was adapted from Khong et al. (2018). A total of 200,000 U-2 OS cells were seeded in 6-well plates containing EtOH-treated coverslips and incubated overnight at 37°C/5% CO_2_. Cells were then untreated or treated with 200 µM arsenite for 2 or 4 h. Subsequently, cells were washed once with PBS, fixed with 4% paraformaldehyde (Santa Cruz Biotechnology; sc-281692) for 5 min, washed three times with PBS, and permeabilized with 0.1% Triton X-100 in PBS for 5 min. Cells were then incubated in buffer A (2× RNase-free saline sodium citrate [SSC], 10% formamide) for 5 min. The coverslips were transferred to a hybridization chamber containing 12.5 µM p21 single-molecule FISH probes (VSMF-2055-5; BioSearch) and incubated overnight at 37°C. After overnight incubation, the coverslips are transferred to a new 6-well plate and washed twice with buffer A with 30 min incubations each at 37°C, washed once with buffer B (2× RNase-free SSC) with 5 min incubation at room temperature, and mounted on slides with VECTASHIELD Antifade Mounting Medium (Vector Laboratories; H-1000).

### Imaging

#### Analysis of individual p-Rb and p21 nuclear intensities

Cells were imaged with the PerkinElmer Opera Phenix using the 40× 1.1-NA water objective (PerkinElmer; HH1400422) at the BioFrontiers Advanced Light Microscopy Core, or Spinning Disk Confocal Nikon Ti-E microscope with a CFI60 Aprochromat Lambda S LWD 40× water immersion objective lens, or a CFI60 Plan Apochromat Lambda 20× objective lens and a 2× Andor Ultra 8888 EMCCD camera. Appropriate lasers, transmission, and exposure times were used to capture images at large dynamic ranges and were kept consistent within each experiment. Images were exported as TIFF files. All images shown are either single *z*-slice or *z*-stacked and altered with Fire LUT, with the help of ImageJ and FIJI plugin, and compiled using Adobe Photoshop and Illustrator.

#### Analysis of stress granules

Cells were imaged using a GE widefield DeltaVision Elite microscope with an Olympus UPlan-SApo 100× 1.40-NA objective oil Objective lens and a PCO Edge sCMOS camera with the help of SoftWoRx Imaging software, or Spinning Disk Confocal Nikon Ti-E microscope with a CFI60 Plan Aprochromat Lambda 100× objective lens and a 2× Andor Ultra 8888 EMCCD camera. Appropriate lasers, transmission, and exposure times were used to capture images at large dynamic ranges and were kept consistent within each experiment. All images shown are *z*-stacked images of entire cells using ImageJ with FIJI plugin and Adobe Photoshop.

#### Analysis of *p21* mRNAs by single-molecule FISH

Cells were imaged using a Spinning Disk Confocal Nikon Ti-E microscope with a CFI60 Plan Aprochromat Lambda 100× objective lens and a 2× Andor Ultra 8888 EMCCD camera. Appropriate lasers, transmission, and exposure times were used to capture images at large dynamic ranges and were kept consistent within each experiment. All images shown are *z*-stacked images of entire cells using ImageJ with FIJI plugin and Adobe Photoshop.

### Image analysis

#### Analysis of individual phospho-Rb and p21 nuclear mean intensities

Exported TIFF files were imported into Imaris 9.8.2 version for image analysis. Nuclear mean intensities for phospho-Rb and p21 were quantified using the surface tool. The surface tool was used to define the nucleus by the DAPI stain. The tool was applied identically for every compared sample. Individual nuclear mean intensities of phospho-Rb and p21 immunostaining were then exported from the surfaces. In Figure 2G, quantification of p-Rb nuclear mean intensities was performed using MATLAB and ImageJ with FIJI plugin custom batch scripts.

#### Analysis of stress granules

Imaging files were imported directly into Imaris 9.8.2 version for image analysis. Stress granules were identified and quantified using the surface tool with PABP1 fluorescence. The tool was applied identically for every compared sample. Stress granule–positive cells from Figure 1 were determined manually by visual inspection.

#### Analysis of *p21* mRNAs by single-molecule FISH

Imaging files were imported directly into Imaris 9.8.2 version for image analysis. *p21* single-molecule FISH spots were identified and quantified using the spots tool. The tool was applied equally to all compared samples. Cells were manually segmented using the surface tool. And fish spots for each cell were then determined by combining spots and surface.

## SUPPLEMENTAL MATERIAL

Supplemental material is available for this article.

## COMPETING INTEREST STATEMENT

R.P. is a cofounder of Illumen Therapeutics and serves on the SAB of Ascidians.

## Figures and Tables

**Anthony Khong:**
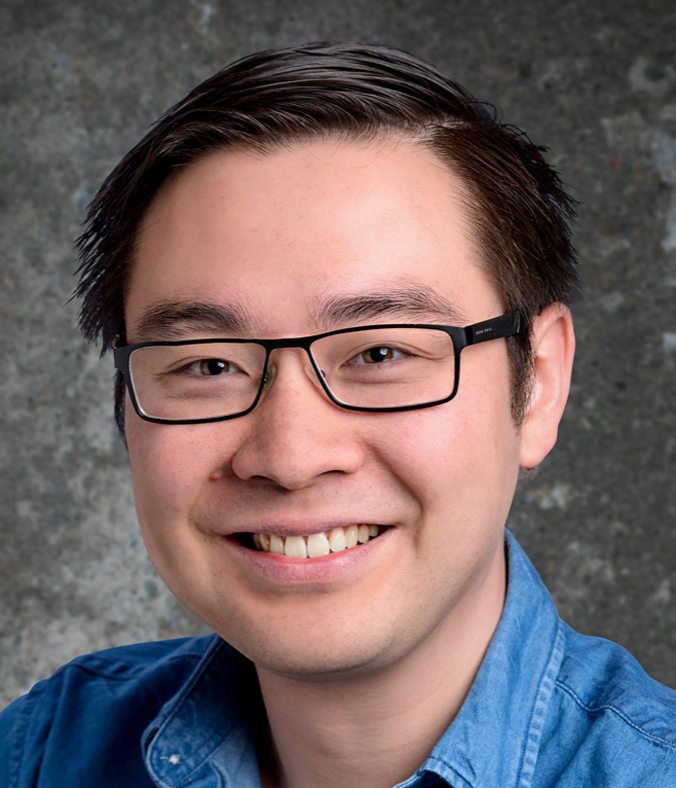


## MEET THE FIRST AUTHOR

***Meet the First Author(s)* is an editorial feature within *RNA*, in which the first author(s) of research-based papers in each issue have the opportunity to introduce themselves and their work to readers of *RNA* and the RNA research community. Anthony Khong is the first author of this paper, “Stress granules promote quiescence by enhancing p21 levels and reducing phospho-Rb.” Anthony is currently an Assistant Professor at the Cancer Science Institute of Singapore and the Department of Physiology at the National University of Singapore. His lab focuses on understanding how cellular stress response pathways shape cancer biology, with particular emphasis on the interplay between the integrated stress response, mTOR signaling, and RNA-driven biomolecular condensates.**

**What are the major results described in your paper and how do they impact this branch of the field?**

Our key finding is that stress granules promote cellular quiescence. This is exciting because it suggests that stress granules may have adaptive roles beyond their previously described functions—such as inhibiting apoptosis and inflammasome activation by sequestering apoptotic proteins, or acting as “plugs” to facilitate lysosomal repair, etc. Regulating the cell cycle is critical for allowing cells to pause, repair damage, and safeguard genomic integrity.

This paper lays the groundwork for future studies to uncover how stress granules drive cell-cycle exit and the consequences of this regulation. In particular, it raises important questions about whether stress granule–mediated quiescence enables cancer cells or tumors to adapt to cancer-associated stresses—areas that my lab is actively investigating.

**What led you to study RNA or this aspect of RNA science?**

RNA has fascinated me throughout my PhD and postdoctoral training, during which I explored RNA virus–host interactions, IRES elements, stress granules, and mRNP organization. My interest began as an undergraduate when I took a course taught by Professor Christopher Proud at the University of British Columbia. I was captivated by his lectures on RNA biology, particularly how cells rapidly modulate gene expression in response to stress through pathways such as the integrated stress response (ISR) and mTOR signaling.

This early curiosity led me to pursue my PhD with Professor Eric Jan, where I studied how viruses circumvent cellular stress response pathways to translate their RNA genomes. Before selecting a postdoctoral lab, I attended a seminar by Professor Roy Parker at RiboWest (Edmonton, 2015), where he presented his lab's groundbreaking work on isolating and characterizing the stress granule proteome (Jain et al. 2016). This discovery was transformative, as it provided a comprehensive picture of stress granule composition and opened new avenues for understanding their assembly and functions. Motivated by these findings—and by Roy Parker's mentorship style—I joined his lab in 2016 to study the principles of stress granule assembly and their biological roles.

**If you were able to give one piece of advice to your younger self, what would that be?**

I would tell my younger self to focus on the things I can control—intellectual growth and skill development. Science is inherently challenging, and experimental success often involves an element of “luck.” It becomes even more difficult if I don't fully understand the scope of the research question (or equally exciting alternative questions that could be explored), the pitfalls of the hypothesis, the limitations of the approaches, how to interpret data, and how to communicate findings effectively. It's easy to feel discouraged when a hypothesis fails or when experiments hit roadblocks, but by prioritizing intellectual growth and building strong skills, I create more opportunities to be “lucky” by quickly refining or shifting hypotheses, generating quality data, and asking better questions.

I emphasize this mindset to my team as well. Students can feel demotivated when experiments fail, but instead of measuring themselves by experimental outcomes, it's healthier and more productive to evaluate their progress through their intellectual growth and skill development.

**Are there specific individuals or groups who have influenced your philosophy or approach to science?**

Many individuals during my PhD and postdoctoral training have contributed to my growth as a scientist, but my postdoctoral mentor, Roy Parker, was particularly instrumental in shaping my scientific approach. Roy often emphasized the importance of intellectual growth and effective communication for the following reasons. First, it is critical to know your field deeply to identify exciting and impactful research questions. Second, having a broad scientific perspective allows one to connect ideas across disciplines and adopt new approaches. Third, effective communication is not just about “selling” one's work or building collaborations—it reflects clarity of thought, which is essential for intellectual development.

These lessons have profoundly influenced my philosophy. I make it a habit to present and write frequently as a means of sharpening my thinking, and I encourage my team to do the same as part of their professional growth.

**What are your subsequent near- or long-term career plans?**

My long-term career goal is to uncover fundamental RNA processes involved in cellular stress responses, particularly those triggered by the tumor microenvironment (e.g., chemotherapeutics, hypoxia, and nutrient starvation). This includes studying translation control, RNA stability, stress granules, and P-bodies under various stress conditions and cancer-related mutations, with the aim of identifying new therapeutic modalities that exploit cancer vulnerabilities during stress.

Over the past two years as a principal investigator, I have also come to deeply value mentoring the next generation of RNA and cancer scientists. Watching students grow intellectually, develop new skills, communicate effectively, and build confidence has been one of the most rewarding aspects of my new role. My goal is not only to advance RNA science but also to foster an environment where students can learn, grow, and succeed.
